# Hypothalamic Deep Brain Stimulation Reduces Weight Gain in an Obesity-Animal Model

**DOI:** 10.1371/journal.pone.0030672

**Published:** 2012-01-25

**Authors:** William P. Melega, Goran Lacan, Alessandra A. Gorgulho, Eric J. Behnke, Antonio A. F. De Salles

**Affiliations:** 1 Department of Molecular and Medical Pharmacology, David Geffen School of Medicine at University of California Los Angeles, Los Angeles, California, United States of America; 2 Department of Neurosurgery, David Geffen School of Medicine at University of California Los Angeles, Los Angeles, California, United States of America; I2MC INSERM UMR U1048, France

## Abstract

Prior studies of appetite regulatory networks, primarily in rodents, have established that targeted electrical stimulation of ventromedial hypothalamus (VMH) can alter food intake patterns and metabolic homeostasis. Consideration of this method for weight modulation in humans with severe overeating disorders and morbid obesity can be further advanced by modeling procedures and assessing endpoints that can provide preclinical data on efficacy and safety. In this study we adapted human deep brain stimulation (DBS) stereotactic methods and instrumentation to demonstrate in a large animal model the modulation of weight gain with VMH-DBS. Female Göttingen minipigs were used because of their dietary habits, physiologic characteristics, and brain structures that resemble those of primates. Further, these animals become obese on extra-feeding regimens. DBS electrodes were first bilaterally implanted into the VMH of the animals (n = 8) which were then maintained on a restricted food regimen for 1 mo following the surgery. The daily amount of food was then doubled for the next 2 mo in all animals to produce obesity associated with extra calorie intake, with half of the animals (n = 4) concurrently receiving continuous low frequency (50 Hz) VMH-DBS. Adverse motoric or behavioral effects were not observed subsequent to the surgical procedure or during the DBS period. Throughout this 2 mo DBS period, all animals consumed the doubled amount of daily food. However, the animals that had received VMH-DBS showed a cumulative weight gain (6.1±0.4 kg; mean ± SEM) that was lower than the nonstimulated VMH-DBS animals (9.4±1.3 kg; p<0.05), suggestive of a DBS-associated increase in metabolic rate. These results in a porcine obesity model demonstrate the efficacy and behavioral safety of a low frequency VMH-DBS application as a potential clinical strategy for modulation of body weight.

## Introduction

According to the U.S. Census Bureau statistics (data for 2006), 34.3% of the adult population in the US is considered obese, as defined by a Body Mass Index (BMI) over 30 kg/m^2^ (www.census.gov). For some individuals, obesity factors result in a condition of morbid obesity (clinically severe; BMI≥40) which affects 5.7% of the population in the U.S. according to national survey data [1]. This condition has many health risk ramifications at both the individual and societal levels that include hypertension, hyperlipidemia, cardiomyopathy, diabetes, hypoventilation disorders, cholelithiasis, degenerative arthritis, and psychosocial impairments [2].

Present treatments to control morbid obesity include a wide variety of drugs and nutritional/dietetic counseling but increasingly only gastrointestinal tract surgical procedures, particularly the Roux-en-Y gastric bypass, have provided successful therapeutic approaches [3], [4]. However, prospective analyses from results of surgical interventions showing undesired side effects and failure of long-term control of the disease in morbidly obese patients (BMI 40–50 kg/m^2^) and super obese patients (BMI>50 kg/m^2^) provide a rationale for exploring other effective modality options [5]–[7]. In particular, it has been suggested that more translational and preclinical research studies be conducted on the potential role of DBS that targets the ventromedial hypothalamus (VMH) and lateral hypothalamus area (LHA) for the treatment of obesity and food intake disorders [8]–[10].

### Human DBS Applications for Obesity

The potential for hypothalamic DBS clinical applications related to control of food intake, fat distribution, and body weight has not been fully explored. Collectively, data from prior animal studies that demonstrated modulation of food intake with electrical stimulation have provided the rationale for a small number of human DBS studies. However, in 2008 and 2010, two case reports described mixed results on the use of hypothalamic DBS to produce weight loss [11], [12] thus highlighting the need for evaluation of DBS delivery approaches that can achieve desired weight modulation without adverse effects. Such studies can be conducted in a large animal model in which human DBS stereotactic methods can be evaluated for efficacy and safety.

### Porcine Models in Obesity and Neuroscience Research

Miniature pigs (minipigs) have gained increasing importance as an alternative non-rodent species to the dog or monkey for basic and applied biomedical research. The pig genome relative to that of the rodent is more closely related to the human genome [13]. Comparisons between the CNS of the rodent and pig show that the pig's gyrencephalic brain resembles the human brain more in anatomy, growth, and development than do the brains of commonly used small laboratory animals [14]. Also, the relatively larger size of the minipig brain allows for identification of cortical and subcortical structures by imaging techniques (MRI, PET, ventriculography), and the use of clinical stereotactic methods and instrumentation [15].

**Figure 1:**
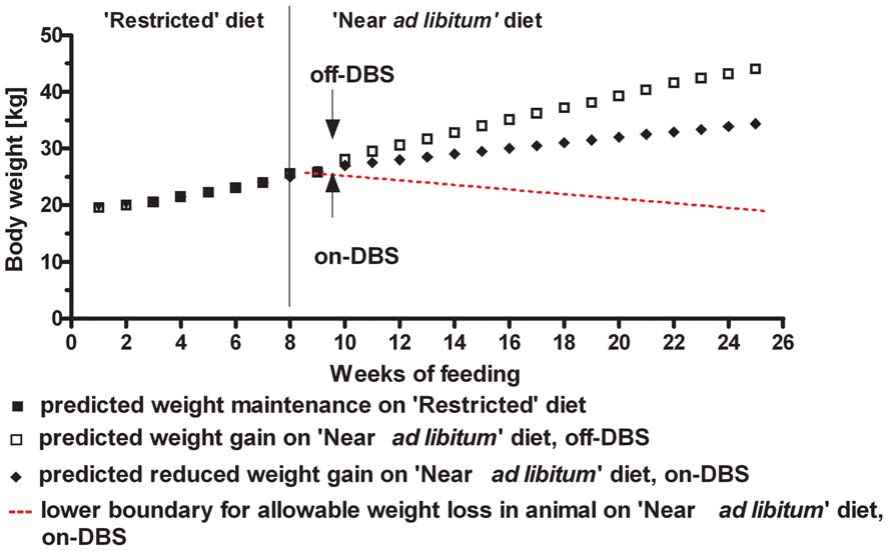
Predicted effect of hypothalamic DBS on weight gain in female Göttingen minipig. Study Design. We hypothesized that low frequency DBS would reduce the weight gain (**♦**) or induce a weight loss (red line), based on prior literature data on weight gain in the Göttingen minipig. Predicted values are also shown for weight changes (▪) on a ‘Restricted’ diet (450 g/day) and for further weight gain (□) on a ‘Near *ad libitum*’ diet (900 g/day).

Rodent models of obesity have provided a wealth of information on basic mechanisms modulating hunger and weight regulation. However, there are several areas where studies in pigs may offer advantages as a model for aspects of human obesity. Göttingen minipigs are similar to humans in digestive physiology, dietary habits, and fat deposition [16]. In the laboratory setting, they can be provided a normative ‘restricted diet’ regimen, (i.e. a defined daily food intake according to animal's age) to maintain a lean healthy phenotype that results in animals with more ‘standardized’ body weight and physiology for a particular age range. This ‘restricted’ diet regimen” pattern (weight gain vs. age) produces a ‘normative’ growth curve as illustrated in Figure 1, that was derived from pooled data for >400 animals from birth through 2 years (data obtained from the supplier, Marshall BioResources, NY).

Minipigs will exhibit hyperphagic behavior when food is provided, *ad libitum*, analogous to the overeating-craving food observed in obese humans. This increase in food intake results in significant weight gain [17], [18] relative to animals on a ‘restricted’ diet. Both male and female Gottingen minipigs become obese, as defined by relative backfat thickness, when fed *ad libitum*, but the females gain significantly more body weight.

Under conditions of food presentation in excess of the ‘restricted’ diet, the Göttingen minipig can be used to model human pathologies linked to obesity [19]–[21] and to assess pharmacologic interventions, as was shown in a study that used liraglutide, a glucagon-like peptide-1 analog [17] to suppress excessive food intake.

In our study, we used female animals and administered a normative ‘restricted diet’ regimen that corresponded to 400–450 g/day of feed for promoting weight maintenance throughout the pre-DBS period. Subsequently, the daily food was doubled to 900 g/day (i.e., ‘Near *ad libitum*’) in all animals for a 2 mo period to establish a controlled condition facilitating obesity, as similarly used and validated in prior studies [22]. During this period, DBS was activated in one-half of the animals, and it was hypothesized that active VMH-DBS would be associated with a reduced weight gain relative to that in off-DBS animals (Figure 1 illustrates the hypothesized effect).

Since low frequency (50–60 Hz) VMH-electrical stimulation negatively modulated weight and food intake in rodent studies, we hypothesized that similar effects would be achieved by low frequency VMH-DBS in a Göttingen minipig model of obesity. In this study, we have shown that stereotactic targeting methods and instrumentation that have been well-established in human DBS applications can be adapted to studies in the Göttingen minipig to demonstrate efficacy of low frequency VMH-DBS for modulation of body weight gain.

## Methods

### Ethics Statement

This study was carried out in strict accordance with the recommendations in the Guide for the Care and Use of Laboratory Animals of the National Institutes of Health, and all procedures were approved by the Office of Animal Research Oversight/Chancellor's Animal Research Committee at the University of California, Los Angeles (ARC# 2010-089-01B). All efforts were made to minimize animal suffering. Surgeries were performed under general anesthesia with analgesic and antibiotic administration.

### Animals

Sexually mature female Göttingen minipigs (Marshall BioResources, North Rose, NY) aged 9–11 months and weighing 20–27 kg were entered into the study.

### Housing and Feeding

The animals were singly-housed within enclosures (1.1×1.8 m) in a dedicated room maintained at 22°C, relative humidity 40%. The enclosures allowed for visual contact among animals. For the complete monitoring of food intake, pre- and post-surgery, the animals were fed once daily between 0730 and 0800 on a ‘Restricted’ diet regimen of 450 g/day (Prolab Mini-pig diet 5P94; PMI Nutrition International, Brentwood, MO, USA), to promote normal weight maintenance without the development of obesity [18], [23]. Water was available *ad libitum*. At one mo after full recovery following the surgery, all animals (n = 8) received for 2 mo a daily doubled-food feeding, 900 g/day, previously referred to as a ‘Near *ad libitum*’ diet regimen [18]. Concurrently, one half of the animals (n = 4) received DBS continuously (see Figure 1 for predicted effect). The animals were weighed on the same day, once weekly, on a mechanical scale (±0.2 kg precision) in the morning prior to their normal feeding time.

### Behavioral Observations

The feeding and behavioral observations were conducted by veterinary and lab personnel blinded to the experimental conditions. The animals in this study (n = 8) were assessed individually at specified times pre-surgery, post-surgery, and during the DBS stimulation period. The motoric and affective behaviors of all animals were observed for ∼4 wks pre-surgery, and subsequently for 4 wks post-surgery. Observations then continued for 2 mo, either on-DBS (n = 4) or off-DBS (n = 4, controls). During 3 d/wk of behavioral testing, the animals were allowed to run along a 15 m long corridor. Five-point Likert scales were used to assess motoric behavior (with 1 indicating normal gait and posture, and 5 indicating pain upon movement and immobility) and affective behavior (with 1 indicating species-typical normal affect, and 5 indicating guarding/aggressive movements). Affective behavior categories consisted of acceptance of petting, willingness to accept and eat small food treats, absence of any guarding response at the site of lead or IPG placement, and resting in a species-typical lateral recumbent position.

### Surgery Procedures

The animals were initially anesthetized with Telazol (4 mg/kg, i.m.) followed by endotracheal intubation. General anesthesia was maintained by inhalation anesthesia (isoflurane 0.5–2.0%). during the MR imaging and surgical procedures. A catheter was placed in the femoral vein for fluid administration. Body temperature, oxygen saturation, pCO_2_, and cardiac rate were monitored continuously throughout the procedures. Following the surgery, the animals were injected with analgesic Carprofen (0.5 mg/kg, i.m.) for 3 d and antibiotic Baytril (2 mg/kg, i.m.) for 5 d, respectively.

### MRI

A stereotactic frame designed for a porcine head [24], [25] was secured with four pins to animal's dorsal skull surface and malar bones (at temporal or zygomatic process) prior to the stereotactic procedures (MRI, DBS electrodes implant). Lidocaine (20 mg/ml) was injected locally at the sites of incisions and of the stereotactic frame attachment to the head. This animal stereotactic frame was designed to fit precisely the human Cosmann-Roberts-Wells (CRW) Radionics stereotactic apparatus (Burlington, MA). A Radionics MRIA-LF localizer (9 axial fiducials) was attached to the frame prior to the MR scan (Siemens Symphony, 1.5 Tesla). The following MR sequences were obtained: Sagittal se T1-weighted imaging (TR 428 ms, TE 8.6 ms, field of view 230 mm, slice thickness 2.0 mm, voxel size 1.0×0.7×2.0 mm); Sagittal mpr-ns T1-weighted imaging (TR 2030 ms, TE 3.01 ms, field of view 300 mm, slice thickness 1.3 mm, voxel size 1.2×1.2×1.3 mm); Coronal tse T2-weighted imaging (TR 2500 ms, TE 32.0 ms, field of view 160 mm, slice thickness 1.2 mm, voxel size 0.8×0.8×1.2 mm); Axial tse T2-weighted imaging (TR 2500 ms, TE 32.0 ms, field of view 160 mm, slice thickness 1.2 mm, voxel size 0.8×0.8×1.2 mm). A coronal reconstruction from the sagittal volumetric sequence was generated and served as the basis for stereotactic planning.

### VMH Targeting

The VMH target was planned according to regional landmarks identified in a published stereotactic brain atlas of the Gottingen minipig species [26]–[29] and the MR images obtained with the attached frame and fiducials, using the iPlan 2.6 software (BrainLab, Feldkirchen, Germany).

Following the stereotactic target planning and target verification on the phantom, the CRW apparatus with target coordinates dialed-in, was attached to the frame affixed to animal's head for the subsequent stereotactic lead implants. After a midline skin incision, the dorsal skull surface was exposed and 1.5 mm diameter holes were drilled bilaterally for the approach to the hypothalamic targets [30]. A polyimide guide tube (O.D. 1.04 mm, I.D. 0.89 mm; MinVasive Components, Trenton, GA) fitted with the stainless steel stylet was inserted into the brain and then the tube was secured to the skull with acrylic adhesive. The distal tip of the guide tube was stereotactically positioned 4 mm above the VMH target. The stylet was removed and the stimulating 4-polar DBS lead was inserted through the guide tube so that its distal tip protruded 4–5 mm from the tip of the guide tube into the targeted area of the hypothalamus. A miniature DBS lead (O.D. 0.64 mm, total length 15 cm, NuMED Inc., Hopkinton, NY) had at its distal end four 90% Pt-10% Ir contacts, 0.5 mm long and spaced 0.5 mm apart.

The accuracy of electrode placement in relation to the set stereotactic targets was ascertained by intraoperative x-ray imaging with cross-hair reticles inserted into the CRW frame. The center of the crosshairs visualized in the image coincided with the center-of-arc point, i.e., the calculated and dialed-in stereotactic target for each VMH. Fluoroscopy allowed for intraoperative corrections of the electrode placement in the ventral-dorsal direction (Z-axis) and also for verification of the spacing between electrodes (2–3 mm) inserted parallel to the midline (Monoplane Philips DSA with intracranial/carotid protocol; 6 frames/s; Philips Medical Systems North America Company, Bothell, WA). This entire lead placement method was used for targeting both left and right VMH. The leads were then secured to the skull with titanium screws and mini-plates (Stryker, Kalamazoo, MI) as was recently suggested in a similar procedure on Göttingen minipigs [31]. A post-implant MR scan was obtained to verify lead placement and trajectory, in situ.

The proximal ends of the bilaterally implanted leads were connected to the extension (Dual 4 Channel Extension #3342; Advanced Neuromodulation Systems (ANS), Plano, TX; a St. Jude Medical Company) which was tunneled subcutaneously to the dorsal part of the neck lateral to the right ear of the animal and connected to the implantable pulse generator (IPG) (Genesis, 8-Channel #3608, ANS, Plano, TX) which was subcutaneously implanted. The extension and IPG were secured in place with non-absorbable sutures. This IPG placement method allowed for unrestricted and free movement of the animal as well as for transdermal access for IPG programming and activation without the need to anesthetize or restrain the animal.

### Deep Brain Stimulation

In our study, we used custom-made DBS leads [30] which have electrodes with a contact area of 0.01 cm^2^ that are significantly smaller than the clinical DBS electrode with a contact area of 0.06 cm^2^. When such small surface area electrodes are used, there is a concern that excess electric charge can be generated at the electrode surface that may potentially damage brain tissue. The relationship between electric charge and electrode surface area is expressed as charge density (microcoulombs/cm^2^/phase), i.e., the quantity of delivered electric charge divided by the surface area.

In prior electrical stimulation and DBS studies in brain, histologically-detected tissue damage or its absence was determined following applications of different combinations of stimulation parameters, [32]. Those studies showed a tissue damage dependency on stimulation parameters. From those studies, a plot of charge density vs charge (where charge is the product of pulse width and current) was used to derive a ‘safety zone’, i.e., combinations of stimulation parameters that did not result in tissue damage. Presently, a charge density of 30 microcoulombs/cm^2^/phase is used as a recommended conservative upper limit for clinical DBS studies [32] with the commercial electrodes of defined geometry, although higher charge densities above that limit are still used (Medtronic Soletra® Neurostimulator Manual).

**Figure 2:**
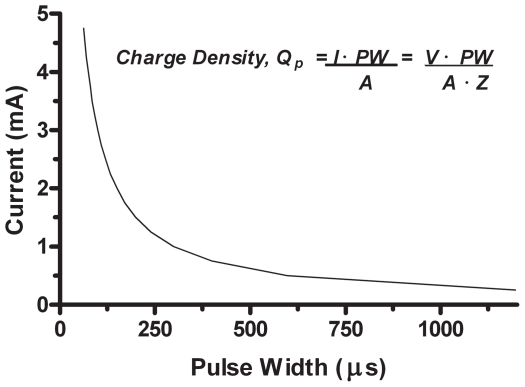
Plot of current vs. pulse width values yielding a charge density (Qp) of 30 µC/cm^2^/phase. For this study, DBS delivery was programmed in constant current mode. To determine initial DBS parameter settings of current (I) and pulse width (PW) that could be presumed not to cause tissue damage, a safety threshold curve was generated from PW and I combinations that yielded a charge density (Qp) of 30 µC/cm^2^/phase – a clinical reference value used as a conservative safety threshold for DBS applications. The equation insert in the graph was used to calculate corresponding PW values for a range of I values, with A = 0.01 cm^2^ for the area of the electrode we used and Qp = 30 µC/cm^2^/phase. Thus, any I and PW combinations to the left of the safety-threshold curve could initially be selected.

Thus, any combination of pulse width, current, and electrode area that yields less than 30 microcoulombs/cm^2^/phase is considered not to result in tissue damage. In Figure 2, we show for our 0.001 cm^2^ electrode area, a curve that represents combinations of pulse width and current resulting in 30 microcoulombs/cm^2^/phase. Accordingly, any combinations to the left of that safety-threshold curve could have been selected. Since we wanted to maintain a low current amplitude for delivering DBS to a relatively small area of the hypothalamus, we initially used a low current (0.5 mA) that was matched with a long PW (507 µs) to achieve a maximal charge density that still remained <30 microcoulombs/cm^2^/phase.

Following the animal's full recovery from surgery (1 mo), the IPG was programmed with DBS parameter settings (amplitude: 0.5 mA; Pulse width: 507 µs; Frequency: 50 Hz) and then activated in a monopolar mode. Contacts 0 and 4, i.e., the most ventral contacts in the left and right VMH leads, were programmed as cathode and the IPG case as anode. Those settings were used for Wks 1, 2, followed by subsequent ramping up of the amplitude: Wks 3–4 1.0 mA; Wks 5–8 1.5 mA. DBS was continuously active throughout the entire 8 wk stimulation period.

### Measurement of Weight and Blood Glucose Levels

In preliminary studies, we observed that the minipigs showed small daily weight fluctuations that were likely due to variable degrees of water and stool retention, as in humans. By design, we delivered DBS to effect a moderate and progressive weight change to achieve a statistically significant ‘long term’ result after a 2 mo DBS stimulation period. The weight measure after that time could then be reliably used to establish the efficacy of the DBS.

After the 2 mo DBS stimulation period was completed, preprandial blood samples were obtained (marginal ear vein puncture) for measurements of blood glucose levels using a glucose oxidase/electrochemical kit (AgaMatrix, Salem, NH).

### Statistics

Our primary outcome measure was cumulative weight change in the 2 mo period under the condition of either on- or off-DBS. Since a directional hypothesis was explicitly stipulated in advance of the study (as shown in Figure 1, i.e., hypothesized weight loss with time), we used a one-tail t-test to determine statistical significance. In further support of the use of a directional hypothesis, the 50 Hz stimulation that we used had been evaluated in numerous prior studies in rodents and resulted in either reduced food intake and/or weight loss [33]–[38]. Presently, there are no reports of weight gain with similar experimental DBS designs using low frequency VMH stimulation, as opposed to high frequency VMH stimulation that we explored in a nonhuman primate study [30].

### Post-Mortem Histology and Immunohistochemistry

After 2 mo of either on- or off-DBS (controls), all animals were euthanized with pentobarbital (100 mg/kg, i.v.). The brains were removed within 20 min; 5 mm coronal blocks were obtained and were frozen for ∼30 s in isopentane maintained at −35°C on dry ice, and then stored at −80°C. For each brain, the block containing the hypothalamus was cut into 20 µm sections on a cryostat (Leica CM 3050) maintained at −20°C (chamber temperature, −15°C cutting head temperature) and thaw-mounted onto gelatin-subbed slides.

Selected sections were post-fixed for 10 min in acetone at −20°C or in 4% paraformaldehyde solution in 0.1 M PBS, pH 7.4, at room temperature for 15 min and then stained with hematoxylin and eosin, and cresyl violet to identify the terminal region of the implanted electrodes. Adjacent sections were then immunostained for GFAP. Briefly, slides with acetone fixed sections were incubated with 0.3% H_2_O_2_ solution in PBS for 10 min at room temperature to block endogenous peroxidase activity, rinsed and preincubated for 1 h in 3% normal horse serum (NHS) in PBS. Sections were then incubated with mouse monoclonal anti-GFAP (Millipore, Temecula, CA), 1∶200, in PBS with 3% NHS for 1 h at room temperature. Following rinses, the sections were incubated with biotinylated horse anti-mouse IgG (1∶200) (Vector Labs, Burlingame, CA) for 1 h and then with ABC (Vector Labs), 1∶200, for 1 h at room temperature. GFAP immunoreactivity was visualized with DAB with nickel ammonium sulfate enhancement. Sections were counterstained with Pyronin Y.

## Results

### Behavioral Observations

The motoric and affective behavior of all animals remained unchanged throughout the pre-surgery, and the post-surgery off-DBS and on-DBS periods. The on-DBS animals showed no adverse reaction to the initial activation of the DBS parameter setting at (0.5 mA, 50 Hz, PW 507 µs) or, subsequently, throughout the DBS stimulation when the amplitude was increased and maintained at 1.5 mA. These conclusions were based on the animals' overt behavior in different circumstances that included being frequently in a recumbent position during the day when observed through a viewing window on the door outside of their cage area, their rolling on their side to accept stroking/petting of their belly area, and maintaining that position for the duration of the interaction.

### Surgery and MRI Procedures

**Figure 3:**
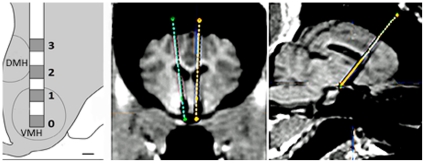
Schematic of DBS lead placement in VMH: Fusion of pre-operative targeting with post-operative MR images. Left: Custom-made DBS leads (O.D. 0.64 mm), were scaled to size for use in the minipig and implanted bilaterally in the VMH regions. The anatomy schematic (adapted from [42] show a coronal hemisection depicting the area of the VMH region occupied by one DBS lead and the relative location of the most distal contact that was used for monopolar stimulation (scale bar = 500 µm). Middle and Right: The pre-operative MR scan was fused to the post-operative MR scan to verify accurate bilateral placement of the 2 DBS leads into the VMH. Images are shown in coronal and sagittal planes. The pre-operative scan was used to plan the DBS lead trajectories which are represented by the dotted lines. The post-operative scan shows the DBS leads (in black) which have distorted volumes due to MR artifact.

A schematic drawing provides an estimate of the actual target area occupied by the custom-designed electrode (Figure 3). The results of previous imaging and histologic studies of Gottingen minipig brain allowed for accurate stereotactic targeting of different brain structures in this species [15], [29], [31], [39]–[41]. Building on these referent results, we defined the VMH target for DBS in the central part of ventromedial hypothalamic nucleus. The ovoid shaped ventromedial hypothalamic area in the Göttingen minipig extends ∼3 mm rostro-caudally between the anterior hypothalamic area and the premammillary nucleus [42]. The largest coronal VMH cross section with our targeted central nucleus has dorsal extent of ∼2 mm and medial to lateral extent of ∼1.8 mm. The stimulating electrode (diameter 0.64 mm) was targeted to be positioned parallel to the midline and ∼1 mm lateral to the third ventricle so that the two most ventral contacts passing through VMH encompassed the whole ventral-dorsal extent of the nucleus. Based on the preoperative MRI scans that were used for stereotactic surgical planning, the targeted central part of VMH had coordinates ∼10 mm anterior to the posterior commissure and ∼8 mm inferior to the AC-PC line at the level of the pituitary stalk between the optic chiasm and the mammillary body.

The iPlan 2.6 software (BrainLab, Germany) was used to derive the bilateral VMH targets, with confirmation assessed either with a post-operative MR scan after the leads were implanted (Figure 3) or with intraoperative fluoroscopy. The trajectories of the electrodes were observed to terminate in the ventral hypothalamic region during the cryostat-cutting of the frozen brain block containing the hypothalamus but could not be visualized in 20 µm sections (data not shown).

The surgery and MRI procedures were unremarkable. The animals well-tolerated the general anesthesia (∼8 h) and they regained consciousness within 2 h after its termination at the end of the surgery. Normal appetite was observed the following day. Prior to initiation of VMH-DBS, we continued the pre-surgery standardized food regimen of 450 g/day for 1 mo post-surgery in order to observe maintenance of pre-surgery weight, affective and motor behaviors, and to allow complete recovery from the surgical procedure-DBS lead implants.

### VMH-DBS Programming Procedures

The position of IPG in the dorsal part of the neck allowed for an easy access for programming and activation transdermally without the need to anesthetize and restrain the animal. Our initial selection of parameter settings (50 Hz, 0.5 mA, and 507 µs pulse width) yielded a charge density below the 30 µC/cm^2^/phase threshold-safety curve, used for clinical DBS applications with commercial electrodes (Figure 3). Those settings were used for first 2 wks of VMH-DBS concurrently with the start of the 900 g/day daily food ration. Since throughout that period a reduction in food intake was not observed, the current was increased to 1 mA for the next 2 wks. After this 1 mo of VMH-DBS, some of the animals showed lesser weight gains relative to the off-DBS animals on the same 900 g/day food ration. Since all animals well-tolerated these initial VMH-DBS parameter settings, the amplitude was further increased to 1.5 mA for 4 additional wks for achieving an effect in all animals. The settings of 50 Hz, 1.5 mA, and 507 µs pulse width resulted in a calculated charge density of 76 µC/cm^2^/phase.

### Post-Mortem Histology and Immunohistochemistry

H&E histology and GFAP immunostaining did not show detectable signs of inflammation or reactive gliosis in the VMH brain region (data not shown).

### Weight Gain Modulation with DBS

**Figure 4:**
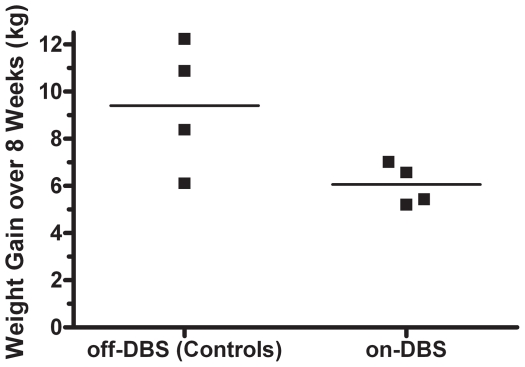
Effects of low frequency VMH-DBS on weight gain in female Göttingen minipigs. After bilateral placement of DBS leads into the VMH followed by a 1 mo recovery period, monopolar stimulation was delivered continuously for 2 mo. The DBS parameters included 50 Hz and 509 µs pulse width with current of 0.5 mA for 2 wks, then 1 mA for 2 wks, and 1.5 mA for 4 wks. For that entire 8 wk period on-DBS, the maintenance ‘Restricted’ daily food ration of 450 g/day was doubled to 900 g/day (the ‘Near *Ad Libitum*’ Diet) for all animals. Both on-DBS (n = 4) and off-DBS (nonstimulated controls; n = 4) animals ate their entire daily food rations. The 8 wk cumulative weight gain for animals on-DBS (6.1±0.4 kg; mean ± SEM) was significantly lower than that for animals off-DBS (9.4±1.3 kg), p<.05).

Throughout the 2 mo of the on-DBS study period, all animals consumed their daily food within 40 min of presentation. After 2 mo, the animals that had received VMH-DBS showed a mean cumulative weight gain of 6.1±0.4 kg (mean ± SEM) that was significantly lower than the 9.4±1.3 kg, measured for the nonstimulated VMH-DBS animals; one-tailed *t*-test, p<.05 (Figure 4).

### Blood Glucose Levels

After the 2 mo DBS stimulation period, preprandial measurements of morning blood glucose levels (after overnight fasting) in on-DBS animals were 3.0±0.3 mM (mean ± SEM) which were not significantly different from the glucose levels of 3.2±0.5 mM measured in control animals. These glucose levels are within the range of mean control values (3–4.1 mM) reported for the Göttingen minipig in prior studies [17], [43], [44].

## Discussion

The rationale for a clinical application of VMH-DBS to treat morbid obesity and severe eating disorders is based on an extensive literature demonstrating the regulation of food intake and satiety by the hypothalamus [45], and the modulation of those functions by hypothalamic electrical stimulation [8], [9].

Consideration of a low frequency VMH-DBS clinical trial can be advanced upon demonstration of its efficacy and safety in preclinical studies in which aspects of the clinical procedures have been modeled. The primary goals of this research were to adapt human neurosurgical methods and DBS instrumentation for use in a large animal obesity model and then evaluate the effects of VMH-DBS on weight modulation and behavior. Our results showed that in the Göttingen minipig under conditions of extra calorie intake, the continuous delivery of low frequency (50 Hz) DBS in the ventral hypothalamus region was associated with a lower weight gain compared to that in animals not receiving DBS.

### Prior Electrical Stimulation Studies in Rodents

In the early 1960's [46], it was recognized that electrical stimulation in the range of 60 Hz of the VMH region of the rat suppressed feeding in animals that were fasted for as long as 5 days. In later studies, VMH stimulation at 50–60 Hz resulted in inhibition of feeding, reduced food intake, and/or reduced weight gain [33], [47]–[49]. Increases in lipolysis were also observed [34] which were attributed to activation of sympathetic pathways to adipose tissues insofar as sympathetic denervation impaired or abolished the electrical stimulation response [35], [36]. Increases in metabolic rate and modulation of energy expenditure were also characterized for this 50–60 Hz range of electrical stimulation [33], [37]. Those results suggested a double effect of electrical stimulation on energy balance. First, it favored a lipolytic effect by the utilization of body fat stored in adipose tissue and that resulted in an anorexigenic effect as characterized by a reduction in food ingestion as a de-novo energy source. Collectively, these studies have been invaluable for defining regional effects of hypothalamic electrical stimulation, albeit with the caveat that most studies were short term (days to weeks) and were conducted in non-obese animals.

### DBS Applications in the Hypothalamus

DBS represents the clinical counterpart of electrical stimulation as used in animal studies. Generally, DBS applications in humans are classified bimodally by frequency range - either high (100–185 Hz) or low (<100 Hz). Within each mode, further options for amplitude (either voltage or current) and pulse width then allow for a wide range of parameter settings combinations. However, in the absence of a detailed theoretical framework for understanding how those variables affect neuronal and glial tissues, the clinician usually uses combinations that follow prior protocols that demonstrated positive clinical outcomes, and then they further modify selected settings empirically to maximize efficacy and minimize adverse effects for individual patients.

Molecular mechanisms underlying the therapeutic effects of DBS remain not well-defined. Initially, experimental evidence supported the theory that high-frequency DBS paradoxically acts like a lesion, i.e., via a neuronal ‘depolarization block.’ However, more recent studies have shown that while high frequency DBS does inhibit somatic activity near the DBS electrode, it can also increase regional output by directly activating axons of local projection neurons. Additionally, surrounding neuropil can be stimulated to different extents as the DBS intensity fades radially from the electrode placement. These ‘secondary’ effects of the high frequency DBS intensity may be analogous to those primarily produced by low-frequency DBS which is hypothesized to activate neurons (cell bodies, axons or both) by enhancing their firing and responsivity to other neural inputs [50].

Since the early 2000's, the posterior hypothalamus has been targeted with high frequency DBS as treatment of cluster headaches [51], facial pain syndromes, and behavior disorders [52], [53]. For example, low frequency (15 Hz) posterior hypothalamic DBS with a relatively low amplitude and long pulse width (0.1–0.4 V, 450 µs) was applied for treatment of drug-resistant aggressiveness in a mentally retarded patient [54]. In contrast, the hypothalamus as a novel target for low frequency DBS in humans has been reported only recently in case reports. Relevant to our preclinical study, hypothalamic DBS effecting changes in body weight in humans was described for a single morbidly obese male [11]. In this case report, weight loss of ∼6% body weight over 5 mo without changes in diet or exercise habits was observed only with low frequency DBS (50 Hz, 3.0–4.0 V, 210 µs) in the VMH area. No such effects were observed with high frequency (130 Hz) over 6 mo. When the low frequency VMH-DBS was turned off, the subject regained the weight he had previously lost, suggesting reversibility of the DBS effect. Another case report on the failure of DBS for weight control in a morbidly obese individual mostly described an adverse effect associated with the procedure [12]. Neither of these case reports explicitly discussed their applied DBS parameters or their postulated effects in terms of weight control.

Here, we attempted to address several of those issues. Firstly, we needed to develop and validate in our lab a large animal model of morbid obesity in which DBS instrumentation and methods could be applied. We greatly benefited from a wide range of prior brain studies in the Göttingen minipig conducted by the Danish group over the last 10 years. In particular, their delineation of the hypothalamus cellular topography clearly indicated its potential applicability for VMH-DBS [42]. Further, studies by Bollen et al. [18], [23], [55] characterized many aspects of the female Göttingen minipig normal physiology and its natural predisposition to become obese with extra calorie intake, thus establishing it as a model for aspects of human obesity. With these prior characterizations of the Göttingen minipig, we readily developed and validated under our lab conditions both maintenance and extra-calorie food intake conditions to be used to test the efficacy of bilateral VMH-DBS for modulating food intake and/or body weight.

Accurate targeting of DBS electrodes to deep structures of the brain, e.g., the hypothalamus, is challenging since small initial trajectory errors are magnified as the depth of insertion increases. Also, transiting across cerebral ventricle membranes enroute to hypothalamus can cause distortion in the trajectory. We significantly obviated those issues with the use of a guide tube to support the minielectrode [30]. The guide tube was implanted with a stainless steel stylet (removed after securing the guide tube) which passed through the trajectory just proximal to the target without mechanical deformation. The electrode was then passed through the guide tube to reach the intended target. Intraoperative fluoroscopy was then used for target confirmation and to make 1–2 mm adjustments in trajectory depth and final positioning (ventral) of the electrode in the target region.

Our prior experience with neurosurgical stereotactic methods, instrumentation and MR imaging for implementing DBS in the human [56]–[58] facilitated their adaptation to the minipig brain. We achieved precise VMH targeting as evidenced by the DBS post-operative MR imaging and post-mortem histology. No significant adverse behavioral effects were observed following the DBS surgery or throughout the DBS stimulation period. The DBS IPG that was subcutaneously implanted in the dorsolateral shoulder region allowed for accessible programming in the conscious and unrestrained animal. Thus, the adaptation of DBS clinical instrumentation (clinical IPG, and connector) could be coupled with an electrode scaled to size for the minipig hypothalamus (electrode diameter: 0.64 mm/animal vs 1.5 mm/clinical).

However, the selection of the initial DBS parameter settings was not evidence-based since the effects of systematic variations of low frequency VMH-DBS stimulation and pulse widths over long periods of time (wks, mo) in any animal species had not been previously described. Accordingly, we selected the stimulation frequency of 50 Hz, based on experimental literature of VMH electrical stimulation showing that a majority of studies with 50 Hz (range: 10–100 Hz) resulted in either a reduction of food intake or an increase in energy utilization. We activated the most distal electrodes with a monopolar configuration to presumably affect a larger VMH region. As our main read-outs on VMH-DBS efficacy were the amount of daily food consumption and weekly weighing, we needed to maintain the same DBS settings for at least 2 wks to determine any significant change. Since our initial DBS settings were without apparent effect for 1–2 wks, we increased the amplitude to 1 mA and then observed a reduced increase in weight (<1 kg) from the previous wk in 2 of 4 animals, suggestive of a DBS effect. Insofar as all animals appeared to well-tolerate the VMH-DBS, we increased the current to 1.5 mA in all animals which was then continued for the second mo of stimulation. This resulted in a charge density of 76 µC/cm^2^/phase. Although that value was higher than the 30 µC/cm^2^/phase limit recommended for clinical applications with the commercial electrodes, it was within a ‘no tissue damage-zone’ derived from multiple non-DBS electrical stimulation and DBS clinical studies [59], [60]. In clinical applications, combinations of low frequency (50–60 Hz) and long pulse width (400–507 µs) will not likely be a safety issue concern insofar as the larger surface area of clinically used DBS electrodes (0.06 cm^2^) yields a charge density within the recommended safety zone.

The present study design with the read-out of weight change did not allow for accurate assessments of alternating short periods of 1–2 wks with on- and off-DBS. However, it was not our intention to induce significant weight fluctuations acutely with DBS since such short term effects may not extrapolate to a safe, long term human application. The overall effect of the VMH-DBS treatment for a 2 mo period of continuous stimulation did result in a significantly lower weight gain of ∼10% relative to weight-matched DBS-off ‘controls’ for the same period of extra-calorie intake. We tentatively attribute this lower weight gain to an increase in metabolic rate insofar as all animals ate their entire daily food ration within 30–40 min of presentation throughout the 2 mo DBS period. This study was not designed to measure changes in metabolic rate, but now having established an effective set of VMH-DBS parameters, future studies employing indirect calorimetry to measure increases in metabolic rate would provide confirmatory data. Further, the use of that method would be ideal for conducting parameter sweeps of DBS settings that can be readily evaluated for their efficacy and reversibility. A VMH DBS-induced increase in metabolic rate has some support from prior experimental and clinical studies. In rodents, electrical stimulation in the hypothalamus resulted in metabolic rate increases [33]–[38]. In humans, a case report showed that a male subject lost weight during VMH DBS without changing his eating habits or his physical activity, suggestive of an increase in metabolic rate.

In conclusion, this study has demonstrated that clinical neurosurgical instrumentation and methods can be applied to preclinical studies in a large animal model. We also demonstrated that DBS leads and electrodes can be scaled down to appropriate size for use in the minipig brain. The DBS that was targeted to the ventral hypothalamus of the Gottingen minipig effected a reduction in weight gain under conditions of extra-calorie intake. These results show that DBS can provide CNS neuromodulation within the hypothalamus and provide preclinical evidence in support for this DBS application as a potential strategy for the treatment of humans with morbid obesity.
